# Comparative Study of the Effect of Pollen Substitute Diets on Honey Bees during Early Spring

**DOI:** 10.3390/insects15020101

**Published:** 2024-02-01

**Authors:** Hyunjee Kim, Olga Frunze, Abdulkadir Yusif Maigoro, Myeong-Lyeol Lee, Jeong-Hyeon Lee, Hyung-Wook Kwon

**Affiliations:** 1 Convergence Research Center for Insect Vectors, Incheon National University, Incheon 22012, Republic of Korea; beamed79@hanmail.net (H.K.); frunzeon@gmail.com (O.F.); aymaigoro@gmail.com (A.Y.M.); be9020@hanmail.net (M.-L.L.); 2 Department of Life Sciences, College of Life Science and Bioengineering, Incheon National University, Incheon 22012, Republic of Korea; jeonghyeon@inu.ac.kr

**Keywords:** honey bee, substitute diet, consumption, brood area, population, colony weight, honey bees weight, *vitellogenin*, deformed wing virus

## Abstract

**Simple Summary:**

Recently, there has been a serious decline in honey bee colonies due to various factors, including habitat loss, diseases, pathogen infections, chemical exposures, and climatic changes. Among these factors, malnutrition-induced stress plays a significant role in colony decline. In our study, we present a novel perspective on maintaining and developing honey bee colonies. Our research demonstrates that a specific pollen substitute diet significantly improves colony performance during early spring, as evidenced by an increase in population, a greater capped brood area, altered colony weight, honey bee weight (dried head + thorax), and *vitellogenin* value. This finding could contribute to enhanced colony maintenance and development during the early spring season, with the potential to prevent colony decline.

**Abstract:**

The nutritional quality of a colony significantly affects its health and strength, particularly because it is required for population growth in the early spring. We investigated the impact of various artificial pollen substitute diets on colony performance in the Republic of Korea during early spring, a critical period for colony health and growth. The colonies were provided with different diets, including the commercial product Megabee (positive control), our developed diet Test A, and four upgraded versions (Diet 1, Diet 2, Diet 3, and Diet 4) of Test A. The negative control group received no supplementary feed. Over 63 days, we observed 24 experimental colonies and assessed various parameters at the colony and individual levels. The results revealed that Diet 2 had the highest consumption and had the most positive impact on population growth, the capped brood area, colony weight, honey bees’ weight, and *vitellogenin* levels. These findings suggested that Diet 2 is most attractive to honey bees and thus holds great promise for improving colony maintenance and development during the crucial early spring period.

## 1. Introduction

Honey bees (*Apis mellifera* L.) primarily rely on nectar and pollen as their sources of nutrition. They use nectar to produce honey, which serves as their primary carbohydrate source [1]. Carbohydrates are the primary component of a honey bee’s diet and supply energy for various functions, including muscular activity, maintaining body heat, and supporting the essential operations of specific organs and glands, such as wax production [2]. Pollen provides a colony with nutritious proteins, lipids, vitamins, and minerals, all of which are required for the growth of broods [3]. Proteins have significant effects on physiological processes (immune response, parasite tolerance and survival, worker lifespan, and drone reproductive quality), brood rearing, adult population development, and royal jelly production [4]. Emerging bees and nurse bees require pollen or protein supplements in their diet that offer an adequate quantity and diversity of amino acids, as certain pollen may lack essential amino acids that bees cannot synthesize themselves and require for their nutritional needs [2]. In 1953, De Groot reported that honey bees require arginine, histidine, isoleucine, leucine, lysine, methionine, phenylalanine, threonine, tryptophan, and valine as essential amino acids [5]. However, the expansion of intensively cultivated landscapes is associated with a decrease in the protein content of beebread, which is fermented from pollen and serves as a primary protein source for honey bees [6]. 

Beekeepers feed supplementary pollen or pollen substitutes during times of pollen dearth to maintain colony strength [7]. The protein-rich substances used in artificial bee diets include soy, pea, yeast, casein, eggs, and microalgae [2,3]. Ideal pollen substitutes should be fully accepted by honey bees year-round, provide comprehensive nutritional support to meet their dietary needs, and be cost-effective [8]. A pollen substitute diet should contain protein, lipids, and other nutritional sources [1,2,8]. A consistent supply of pollen or a protein-supplemented diet can encourage brood raising and promote colony expansion [7]. However, adult workers who are nutritionally stressed, such as larvae, might experience weight loss, shorter survival, less foraging activity, and more aggressive dance behavior [9]. Many beekeepers commonly use commercial or artificial diets to compensate for apparent nutritional deficiencies [2,8].

Several studies have explored the effects of different feeding diets on honey bees. Vg is a yolk protein that is extensively produced in the fat body, released into the hemolymph, and subsequently incorporated by oocytes during development [10]. It is also absorbed in nurse bees’ hypopharyngeal glands, serving as an amino acid source for the production of royal jelly [11]. These studies assessed changes in vitellogenin (Vg) levels in the hemolymph to various types of pollen diets. Differences in these responses depended on the diet during early spring. However, it is important to note that these studies did not evaluate colony-level performance [12]. In another study, honey bee diets, including both pollen-mixed diets and diets with no pollen, were evaluated. Diet consumption, the number of frames, the honey bee head + thorax weight, Vg levels, and the gut microbiota were measured [3]. However, there was no significant effect on Vg expression in the diets examined. The quantity of deformed wing virus (DWV) is used to determine colony strength and is considered an adverse indicator of honey bees’ fitness [13]. One of the most common and pervasive viral infections of *A. mellifera* in the world is DWV [14]. In newly emerged honey bees, DWV infection leads to morphological abnormalities (such as deformed wings) and early death [15]. A significant correlation exists between the DWV titer and colony loss during overwintering [16]. These studies also analyzed the nutritional content of the diets they consumed. In our previous study, we conducted similar nutritional content analyses on artificially developed diets with commercial products and beebread [17]. Our development diets had comparable nutritional content to the commercial diet but a higher total free amino acid content. Subsequently, in the present study, we assessed the colony performance of our developed diets at both the colony and individual levels.

In temperate climates, honey bees exhibit an annual brood-rearing cycle, starting in early spring and rising until summer, following nectar flow, with a significant increase in spring before reaching its peak and a subsequent gradual decrease in late summer, leading to virtual cessation in late fall. The spring season is a time when the colony size expands [18].

This study aimed to assess the impact of various artificial diets on honey bee colonies in Gangneung, Republic of Korea, during the early spring season. Our focus was directed toward formulating a pollen substitute diet to mitigate the depletion of natural pollen resources [19]. No protein supplemental food provided to honey bees can fully substitute for natural pollen in terms of nutritional completeness [2]. In the colony-level assessment, we monitored population size, capped brood area, colony weight, consumption, and preferences. At the individual level, we measured honey bee weight, the expression level of the nutrient-related *Vg* gene, and the presence of the deformed wing virus pathogen. The findings of this study could lead to the development of honey bee diets for beekeepers, facilitating more efficient colony management during the early spring.

## 2. Materials and Method

### 2.1. Preparation of the Pollen Substitute Diet and Experimental Design

Our developed pollen substitute diets included Test A, Diet 1, Diet 2, Diet 3, and Diet 4. The nutritional contents (free sugar, organic acids, free amino acids, inorganic ions, total polyphenols, vitamin C, and pH) of the diets were previously analyzed [17]. Test A is the diet we initially developed. Diet 1, Diet 2, Diet 3, and Diet 4 are upgraded versions from Test A, adding some ingredients. The detailed components and concentrations of each diet are listed in Table 1. To enhance the nutritional quality of Test A, we incorporated four distinct functional additives: soytide (CJ Global Food and Bio Company, Seoul, Korea), apple juice (Jaan Company, Dubai, United Arab Emirates), chlorella powder (Cheonil Herbal Medicine), and mint powder (based on personal communications). In Diet 1, soytide was incorporated as a functional additive because it is characterized by a high protein content and low antinutritional and allergenic properties, facilitating development and nutrient utilization [17,20]. Diet 2 included apple juice as a functional component due to its association with extended longevity in adult codling moths, *Cydia pomonella* [21]. Chlorella powder was introduced into Diet 3 as a functional supplement and is known to influence colony formation and metabolism in honey bees by enhancing fat deposition and *Vg* transcript levels [22]. For Diet 4, mint powder was included as a functional additive because of honey bees’ preference for mint, which can affect their body weight and has been observed to increase weight in broilers fed a peppermint-supplemented diet [23]. 

**Figure 1:**
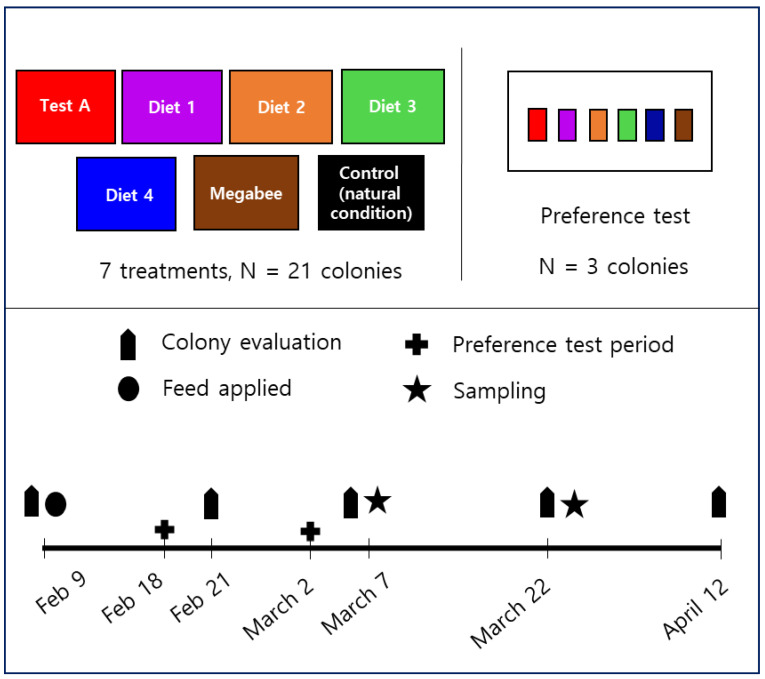
Schematic overview of the experimental design. In the preference test, the name of each diet corresponds to the same color, indicating the same diet. For instance, the red-colored square signifies Test A.

The field experiment was conducted with twenty-four honey bee colonies in Gangneung, Republic of Korea, from 9 February to 12 April 2022 (Figure 1). Each experiment was conducted with three replications. Five artificial diets (Test A, Diet 1, Diet 2, Diet 3, and Diet 4) were tested and a commercial diet, Megabee (Castle Dome Solutions, Helena, AR, USA), which is a blend of plant-based proteins devoid of pollen, widely employed by beekeepers in the U.S., served as the positive control [9]. Additionally, natural conditions in which no protein was added to the diet were used as a negative control. Approximately 1200 g of diet was used to assess the influence of the diet on the honey bee population, capped brood area, colony weight, diet consumption, diet preference, honey bee weight, *Vg*, and *deformed wing virus* (*DWV*) gene expression levels over 63 days.

### 2.2. Consumption

Approximately 1200 g of one diet was applied to the upper side of each colony. Three replications were conducted for each diet. The quantity of supplemental diet consumed was determined by calculating the difference between the initial weight of the supplemental diet and the weight of the diet that remained (g per colony) (consumption = initial diet weight − final diet weight. Consumption was measured at three different times (21 February, 7 March, and 22 March 2022) during the entire experiment. 

### 2.3. Preference

A 400 g portion of each diet was wrapped in vinyl and labeled individually. The six prepared diets were placed on the upper side of the frames in each colony for a duration of 16 days (from 18 February to 2 March) in random order. The same calculation method, as described in Section 2.2, was applied every three days. Finally, the most reduced diet among the tested six diets was associated with the greatest preference. The preference test was conducted using three replications.

### 2.4. Honey Bee Population

The honey bee population was determined by the number of adult bees covering the frame within a hive. Estimation was repeated three times during the entire experiment. Additionally, we calculated the difference in the number of combs covered with adult bees from the initial measurement in each colony. This calculation was prompted by the absence of normalization of the initial honey bee population before initiating the experiment. Based on prior assessments, we assumed that each fully covered frame on both sides contained a population of two thousand honey bees [24].

### 2.5. The Capped Brood Area 

We employed the Puchta method to measure the capped worker brood area approximately every 21 days [25,26]. The capped brood area was determined using the formula S = 3.14 × A/2 × a/2, where S represents the area, A is the length of the ellipse’s long axis, and a is the length of the ellipse’s short axis. We estimated the change in the capped brood area from the initial measurement within the colony. 

### 2.6. Colony Weight

The colony weight was measured by carefully moving the entire hive onto an electronic balance. Measurements were taken before the start of feeding and after feeding three times during the experiment. Each of the colonies was fed with each diet for 42 days (until 22 March), which was before the commencement of foraging activity.

### 2.7. Honey Bees’ Weight Measurements 

To measure honey bee weight, we dissected three honey bees from the field samples over dry ice, distinguishing between the head, thorax (excluding legs and wings), and abdomen. We separately combined the individuals in each group into groups of three heads, three thoraxes, and three abdomens. The weights of the heads and thoraxes were determined by drying them in an oven at 60 °C overnight. 

### 2.8. RNA Extraction and Quantitative PCR (qPCR)

A representative fraction of the colonies was sampled, with 20 honey bees per colony, to obtain *Vg* measurements on 7 March. *DWV* expression levels were analyzed on 7 March and 22 March (*n* = 3 colonies per diet treatment). We sealed all the samples in 15 mL conical tubes using vacuum sealing, then rapidly froze them on dry ice and stored them at −80 °C for subsequent analysis. To extract RNA, we isolated total RNA from the pools of the three abdomens using a Qiagen RNeasy Mini Kit (#74104; Qiagen, Valencia, CA, USA). The total RNA concentration and purity were quantified using OD260/OD280 values between 1.8 and 2.0. We assessed the gene expression levels of *Vg* and *DWV* through quantitative PCR (qPCR) using cDNA templates derived from total RNA. To synthesize cDNA, 1 μg of total RNA was used in conjunction with oligo-dT and the Invitrogen Superscript III enzyme (Grand Island, NY, USA). qPCR was conducted using the StepOne Plus system from Applied Biosystems (Foster City, CA, USA) and SYBR GreenqRT-PCR Master Mix from Fermentas (Burlington, ON, Canada) under the following conditions: initial denaturation at 95 °C for 5 min, 40 cycles of denaturation at 95 °C for 30 s, annealing at 60 °C for 30 s, and extension at 72 °C for 30 s. Detailed information about the qPCR primers can be found in Table S1. The obtained results were standardized against the validated control gene, *β-actin*, utilizing the 2^−ΔΔCt^ method [25]. Each biological replicate was subjected to technical triplicate testing.

### 2.9. Statistical Analyses

We conducted the data analysis using SPSS version 25 (IBM). To identify significant differences among means at a significance level of *p* < 0.05, we employed Duncan’s multiple range tests. The results are presented as the mean values accompanied by their standard deviations (SDs) and standard errors of the means (SEMs). Graphs were created using GraphPad Prism software (version 7.03, Inc., San Diego, CA, USA).

## 3. Results 

### 3.1. Consumption and Preference

**Figure 2:**
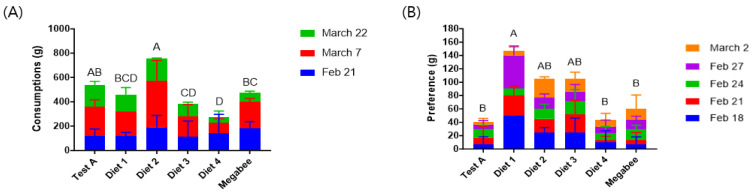
Consumption and preference of different kinds of diets in early spring (g/colony). (**A**) Consumption; (**B**) preference. The means followed by different letters are significantly different according to Duncan’s multiple range comparisons (DMRTs) (*p* < 0.05) (mean ± SD, *n* = 3).

Significant differences were observed in the consumption of various diets over 42 days (*p* < 0.05) (Table 2). Honey bees consumed significantly greater amounts of Diet 2 (758.00 ± 270.00 g), followed by Test A (539.50 ± 28.99 g), Megabee (475.67 ± 45.96 g), Diet 1 (459.67 ± 2.12 g), Diet 3 (385.33 ± 214.50 g), and Diet 4 (276.50 ± 2.12 g) (Figure 2A). Among our developmental diets, the Diet 2 and Test A groups consumed significantly more food than the Megabee group. Regarding preference, significant differences were shown among the various diets over 16 days (*p* < 0.05). Among the diets, Diet 1 (145.00 ± 7.07 g) had the highest consumption, followed by Diet 2 (100.00 ± 0.00 g), Diet 3 (96.67 ± 66.58 g), Megabee (60.00 ± 34.64 g), Diet 4 (53.33 ± 20.82 g), and Test A (40.00 ± 26.46 g) (Figure 2B). Among our developed diets, Diet 1, Diet 2, and Diet 3 produced significantly greater preferences than Megabee.

### 3.2. Colony Population

**Figure 3:**
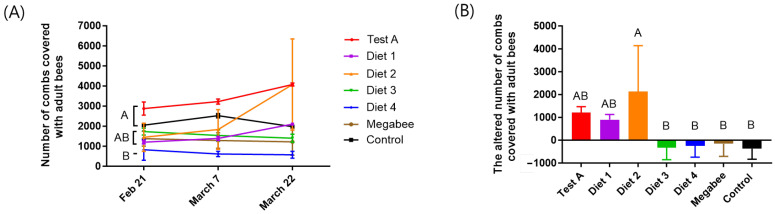
The number of combs covered with adult bees depends on the different diets. (**A**) The number of combs covered with adult bees according to the type of diet consumed at different times (mean ± SEM, *n* = 3). (**B**) The altered number of combs covered with adult bees in early spring. The means followed by different letters are significantly different according to Duncan’s multiple range comparisons (DMRTs) (*p* < 0.05) (mean ± SD, *n* = 3). The same color in (**A**,**B**) indicates the same diet.

The population size exhibited significant differences on 21 February depending on the diet (Figure 3A). Consequently, we analyzed the mean increase in population according to diet from 21 February to 22 March. The results indicated significant statistical differences between the diets (*p* < 0.05) (Table 1). The population of bees in the Diet 2 treatment (2135.00 ± 2001.11 bees) exhibited the greatest increase, followed by those in the Test A (1216.67 ± 251.66 bees) and Diet 1 (900.00 ± 233.02 bees) groups. The Control group (−375.00 ± 459.62 bees) exhibited the most substantial decrease in population, followed by the Diet 3 group (−330.00 ± 523.26 bees), the Diet 4 group (−250.00 ± 494.97 bees), and the Megabee group (−160.00 ± 545.25 bees) (Figure 3B). Among the various diets, Test A, Diet 1, and Diet 2 were significantly greater than those of Megabee and the control in terms of colony population. 

### 3.3. Capped Brood Area

**Figure 4:**
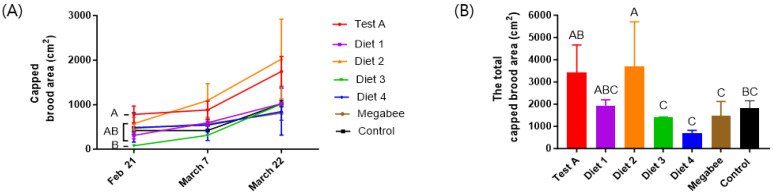
The capped brood area depends on the different diets. (**A**) The capped brood area according to the type of diet consumed at different times (mean ± SEM, *n* = 3). (**B**) The capped brood area during the experiment in early spring. The means followed by different letters are significantly different according to Duncan’s multiple range comparisons (DMRTs) (*p* < 0.05) (mean ± SD, *n* = 3). The same color in (**A**,**B**) indicates the same diet.

The capped brood area showed significant differences on 21 February (Figure 4A). The capped brood area increased during the experimental period in all treatment groups. The total capped brood area exhibited statistically significant differences between the groups fed the different diets (*p* < 0.05) (Table 1 and Figure 4B). Diet 2 (3699.00 ± 2003.83 cm^2^) demonstrated the highest total capped brood area, significantly surpassing Test A (3416.00 ± 1245.64 cm^2^), Diet 1 (1931.00 ± 265.62 cm^2^), Control (1812.00 ± 339.15 cm^2^), Megabee (1483.00 ± 637.01 cm^2^), Diet 3 (1411.00 ± 4.44 cm^2^), and Diet 4 (683.00 ± 131.55 cm^2^). Among our developmental diets, Test A, Diet 1, and Diet 2 had significantly larger total capped brood areas than Megabee and the control. 

### 3.4. Colony Weight

**Figure 5:**
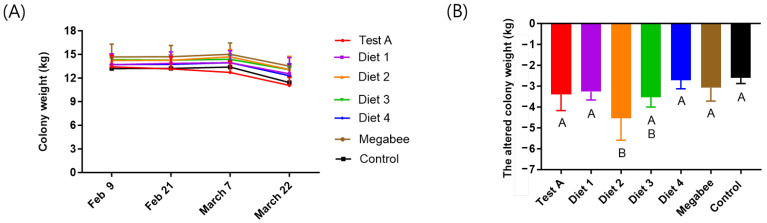
The colony weight depends on the diet. (**A**) Colony weight according to diet type at different times. (**B**) The altered colony weight during the experiment in early spring. The means followed by different letters are significantly different according to Duncan’s multiple range comparisons (DMRTs) (*p* < 0.05) (mean ± SD, *n* = 3). The same color in (**A**,**B**) indicates the same diet.

The colony weight decreased during the experimental period in all treatment groups (Table 1 and Figure 5A). The total colony weight was statistically significantly different between the diet groups (*p* < 0.05) (Figure 5B). Diet 2 (−4.50 ± 1.06 kg) showed the highest decrease in the colony weight followed by Diet 3 (−3.53 ± 0.47 kg), Test A (−3.39 ± 0.78 kg), Diet 1 (−3.27 ± 0.40 kg), Megabee (−3.07 ± 0.64 kg), Diet 4 (−2.72 ± 0.41 kg), and Control (−2.61 ± 0.27 kg). Among our developed diets, the colony weights of the Diet 2 and Diet 3 groups were significantly greater than those of the Megabee and Control groups. 

### 3.5. Honey Bees’ Weight

**Figure 6:**
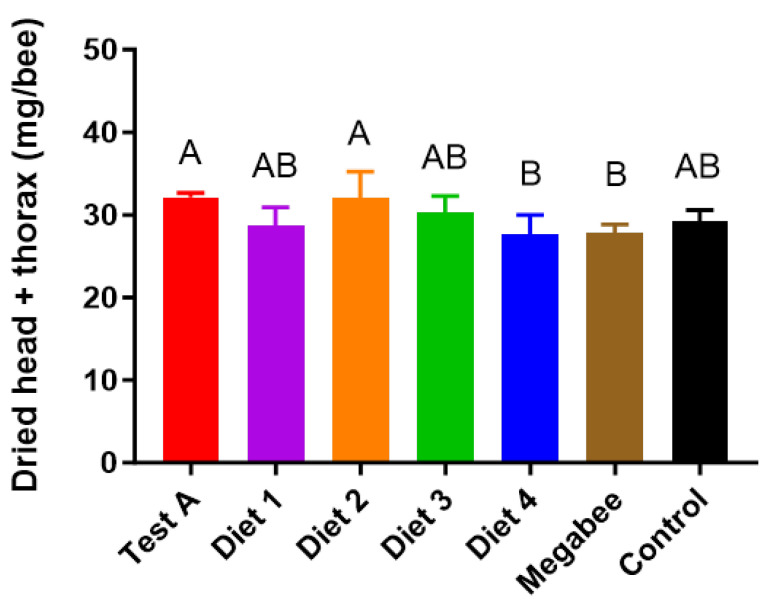
Dried head and thorax weights of different kinds of diets in early spring. The sampling occurred on 22 March. The means followed by different letters are significantly different according to Duncan’s multiple range comparisons (DMRTs) (*p* < 0.05) (mean ± SD, *n* = 3).

There were significant differences in the dried head + thorax weight according to the diet (Figure 6). Test A (32.1 ± 0.51 mg/bee) and Diet 2 (32.00 ± 3.22 mg/bee) exhibited the highest dry head + thorax weights, followed by Diet 3 (30.20 ± 2.04 mg/bee), Control (29.20 ± 1.35 mg/bee), and Diet 1 (28.80 ± 2.14 mg/bee). Megabee (27.80 ± 1.02 mg/bee) and Diet 4 (27.70 ± 2.36 mg/bee) had significantly lighter dried head + thorax weights.

### 3.6. Vitellogenin

**Figure 7:**
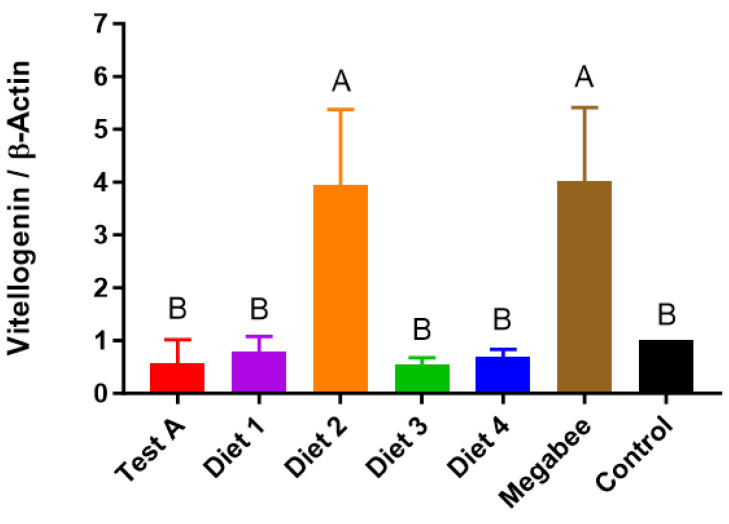
The response of honey bee *vitellogenin* gene expression to different diets in early spring (March 22). The means followed by different letters are significantly different according to Duncan’s multiple range comparisons (DMRTs) (*p* < 0.05) (mean ± SD, *n* = 3).

The *Vg* gene expression of honey bees exhibited significant differences depending on the diet (Figure 7). Megabee- and Diet 2-fed honey bees presented the highest *Vg* gene expression, which was approximately four times greater than that of the control. 

### 3.7. DWV

**Figure 8:**
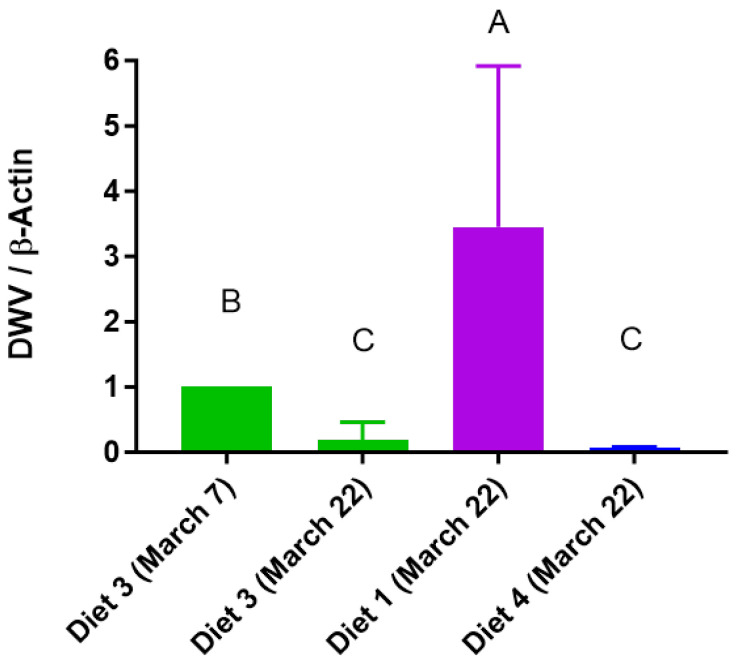
Relative mRNA expression of deformed wing virus (DWV). The means followed by different letters are significantly different according to Duncan’s multiple range comparisons (DMRTs) (*p* < 0.05) (mean ± SD, *n* = 3).

Only the Diet 3-fed honey bees exhibited *DWV* gene expression on March 7 among the treatment groups, but this expression decreased on March 22 (Figure 8). The Diet 1-fed honey bees displayed the highest DWV expression levels on both 7 March and 22 March. 

## 4. Discussion

To address deficiencies in available nutritive pollen forage in the environment, apiculturists often supplement honey bee colonies with artificial and commercial diets. This supplemental feeding is known to stimulate brood production, facilitating the expansion of colony populations [24,25,27]. The nutritive value of the diet significantly influences the development, reproduction, and overall productivity of honey bees [28]. Fluctuations in the nutritional value of natural flora, influenced by changing seasons and the impact of climate change, can have consequences on colony development [29]. The experimental findings presented in this study provide valuable insights into the colony response (consumption, preference, colony population, capped brood development, and colony weight) and the individual honey bee response (head + thorax weight, *Vg* and *DWV* gene expression) to various pollen substitute diets. 

Sugars, amino acids, and lipids are recognized as nutritionally valuable elements that enhance the appeal of a diet to diverse mutualistic animals, with the recent identification of additional appealing constituents, such as volatile organic compounds [30]. Consumption is a key indicator for understanding the quality and impact of a diet. It can be influenced by multiple cues (from the colony and from the diet). In this study, the colony size could not be normalized prior to the experiment. There was a significant difference in the number of honey bees on 21 February among the diets. Test A and Control had the highest number of honey bees. However, the Diet 2 treatment had the greatest increase in the number of honey bees during the experimental period, which indicated that the Diet 2 treatment positively influenced colony development. According to the consumption analysis, the highest consumption was observed in the Diet 2 treatment group, marking the initial attempt to integrate apple juice into the honey bee diet. Given this observation, it is pertinent to delve into the specific components within apple juice that attract honey bees. A comprehensive analysis of the sugar content across 103 apple cultivars revealed that fructose exhibited the highest concentration [31]. These findings led to the hypothesis that the appeal of Diet 2 to honey bees may be attributed to its elevated fructose content. In our previous nutritional analysis, Diet 2 presented a slightly greater fructose content than the other diets [17]. In the literature, honey bees have shown a preference for foraging on apple pollen [32]. Insects recognize food through a combination of behavioral responses, sensory mechanisms, and the perception of host-released odors [33]. The sensory system relies on visual, olfactory, and gustatory cues [33]. Moreover, the behavioral aspect involves experience-based learning and orientation mechanisms, all of which contribute to the identification of food sources [33]. Honey bees have 10 gustatory receptor genes in their genome. Among them, AmGr1 exhibits broad responses to sugars such as sucrose, glucose, trehalose, and maltose [34]. AmGr2 serves as a coreceptor for AmGr1. Moreover, AmGr3 responds specifically to a particular fructose [34]. These findings suggested that fructose has significant nutritional value for honey bees, but further detailed research is needed. However, our understanding of the molecular mechanism responsible for the species-specific feeding preferences of insects is limited. Further in-depth investigations may be warranted to validate and explore this observation. 

In another study, a strong correlation was observed between diet consumption and changes in brood area and adult population size [24]. In the present study, Diet 2, which had the highest consumption of colonies, also had the largest brood area and adult population. These findings suggested that this particular diet fosters the overall development of the colony, particularly in the early spring season. Furthermore, diet consumption plays a crucial role in the conversion of honey bees to Vg, the most abundant nutrient storage molecule in honey bees, which is subsequently transformed into brood feed in the form of royal jelly [12]. Notably, the Diet 2-fed honey bees exhibited the highest *Vg* gene expression levels. Therefore, among the colonies produced from the tested diets, those produced from the Diet 2 treatment had the highest brood area and adult population. Preference analysis further supported these findings, with Diet 1 emerging as the most preferred diet among the options (three times more favored than Test A), followed by Diet 2 and Diet 3. The nutritional value and taste of Diet 1, Diet 2, and Diet 3 likely contributed to their higher preference scores. It has been established that honey bees exhibit a preference for pollen sources rich in both protein and lipids [29,35]. Honey bees might also consider nutritional value when making diet choices. In our previous study, the preferred diets, Diet 1, Diet 2, and Diet 3, had slightly greater total amino acid contents than the other tested diets. Additionally, Diet 1 exhibited the highest total inorganic ion content among the tested diets [17]. Further confirmation is necessary to validate the actual role of specific nutritional components in honey bee health and to understand their physiological contributions to honey bee health. Colony population dynamics revealed significant differences among the diets, emphasizing the role of nutrition in colony growth. Among the diet groups, the Diet 2 group exhibited the most pronounced increase in population density. These findings indicate that these diets positively influence brood development and subsequent population expansion as the new generation of honey bees grows well. In contrast, Control-, Diet 3-, Diet 4-, and Megabee-fed honey bee colonies showed a decrease in population size, suggesting suboptimal dietary conditions for colony growth. In other studies, various pollen substitute diets were associated with distinct patterns of adult population growth [24,28]. The expansion of the capped brood area across all treatment groups is an encouraging sign of successful brood development. The Diet 2-fed colony had the highest total capped brood area, reflecting its positive impact on brood rearing. The study demonstrated that differences in the nutritional composition of diets, coupled with the digestibility and nutrient accessibility to worker bees, significantly influence potential brood production [24]. This implies that in the limited floral resources period, pollen substitute diets offer ample nutrients to sustain brood rearing in colonies. Other studies, conducted with colonies placed under conditions of pollen scarcity, revealed that those colonies receiving supplements demonstrated an improved capacity for brood rearing compared to the control colonies [7,8,36,37]. The brood-rearing capacity of a colony is directly related to the availability of nutrients.

During the experimental period, the colony weight decreased across all the groups. Notably, Diet 2 had the most significant reduction in colony weight. This weight loss might indicate increased consumption due to the expanding population. Notably, the absence of flowering near our experimental apiary, as flowering had not yet begun, resulted in limited access to pollen sources for honey bees. Consequently, honey bees primarily rely on the provided food sources within the colony for consumption. Another study revealed a reduction in colony weight from February to the end of March [38]. 

Examining honey bee weight revealed variations in the dried head + thorax weights across the diets. Notably, Test A and Diet 2 exhibited the most substantial head + thorax weight gain, suggesting their favorable impact on the body development of individual honey bees. This observation aligns with the notion that a worker’s weight accurately mirrors nutritional allocation during its larval stage [39]. Greater head and thorax weights reflect enhanced development of head glands and increased flight muscle mass, which are essential attributes for the fitness of a colony [3]. Additionally, several studies have pinpointed a positive correlation between dry weight and lifespan [5,39]. 

Vg affects the worker sucrose response, foraging start, foraging choice, the caste differentiation process, protection from oxidative and immune attacks, and longevity [22,40,41,42]. The concentration of Vg, a well-known zinc (Zn) carrier, strongly correlates with the hemolymph Zn titer, and its hypothesized role in protecting winter bees from oxidative damage, due to Zn’s known defense against oxidative stress, suggests potential lifespan-extending properties [18]. Adequate nutrient availability is essential for producing Vg, and the levels present in circulation are strongly linked to the intake of protein [43,44]. Therefore, Vg is considered an indicator of honey bee health. In the present study, *Vg* gene expression levels were significantly different according to diet, with Megabee and Diet 2 inducing the highest expression levels. These findings indicate the potential of these diets to stimulate honey bee health. A positive correlation was recorded between the fat body expression levels of *Vg* and *insulin-like peptide 1*, a nutrient sensor gene in worker honey bees [44]. In other studies, the levels of *Vg* gene expression varied based on dietary nutrition, with honey bees fed pollen-rich diets showing higher *Vg* gene expression than those fed pollen-free diets [36,45]. 

We investigated the impact of diet on DWV titers. The expression patterns of DWV varied among the diets; notably, Diet 3 was detected only on March 7, but its presence decreased by March 22. These findings suggested that Diet 3 might contribute to enhanced resistance against DWV. Among our diets, except for Diet 3, Diet 1, and Diet 4, it can be presumed that the feeds in which DWV was not detected among those used in the experiment may be resistant to DWV. The nutrition of adult honey bees enhances larval nutrition and contributes to disease resistance in colonies [46]. This implies that nutrition can influence an individual’s health and susceptibility to disease. However, further investigations are needed to determine the potential implications of these expression patterns for colony health. In the future, it will be necessary to compare honey bee diets containing mixed additive ingredients and assess their impact on colony performance during both the early spring and the fall seasons. 

## 5. Conclusions 

Our study highlights the intricate relationships between various pollen substitute diets and honey bee performance indicators. Diet 2 demonstrated the highest consumption by honey bees, which led to an increased number of honey bees, capped brood area, colony weight, head + thorax honey bee weight, and *Vg* gene expression; notably, DWV infection was not detected in colonies fed Diet 2. The observed trends emphasize the pivotal role of the pollen substitute diet in sustaining honey bee health and colony development. Further research is warranted to unravel the underlying mechanisms driving these effects and their long-term implications for honey bee colonies in various environmental contexts.

## 6. Patents

We have patents related to these diets in the present study, titled ‘Bee Feed Composition’, granted on 12 December 2022 (No. 10-2022-0166677).

## Figures and Tables

**Table 1:** Composition of pollen substitute diets with constant ingredients (%).

Ingredients	Test A	Diet 1	Diet 2	Diet 3	Diet 4
Brewer’s yeast	39.69	39.69	39.69	39.69	39.69
Egg yolk	2.21	2.21	2.21	2.21	2.21
Defatted soybean powder	2.21	-	2.21	2.21	2.21
Sugar	35.36	35.36	35.36	35.36	33.86
Boiled water	15.16	5.16	7.16	5.16	5.16
Canola oil	1.01	1.01	1.01	1.01	1.01
Cellulose	0.88	0.88	0.88	0.88	0.88
Wheat bran powder	0.88	0.88	0.88	0.88	0.88
Multiple vitamins	0.44	0.44	0.44	0.44	0.44
L-methionine	0.10	0.10	0.10	0.10	0.10
L-lysine	0.24	0.24	0.24	0.24	0.24
Citric acid	1.85	1.85	1.85	1.85	1.85
IMP	-	0.00	0.00	0.00	0.00
GMP	-	0.00	0.00	0.00	0.00
Tangerine juice	-	10.00	4.00	10.00	10.00
Soytide powder	-	2.21	-	-	-
Apple juice	-	-	4.00	-	-
Chlorella powder	-	-	-	0.08	-
Mint powder	-	-	-	-	3.00

IMP means inosine-5′-monophosphate, and GMP means guanosine-5′-monophosphate.

**Table 2:** The effect of different pollen substitute diets on colony levels in the early spring (mean ± SD).

Diets	Consumptions (g/Colony)	Preference (g/Colony)	Populations (Bees/Colony)	Total Capped Brood Area (cm^2^)	The Altered Colony Weight (kg)
Test A	539.50 ± 28.99 ^ab^	40.00 ± 26.46 ^b^	1216.67 ± 251.66 ^ab^	3416 ± 1245.64 ^ab^	−3.39 ± 0.78 ^a^
Diet 1	459.67 ± 2.12 ^bcd^	145.00 ± 7.07 ^a^	900.00 ± 233.02 ^ab^	1931 ± 265.62 ^abc^	−3.27 ± 0.40 ^a^
Diet 2	758.00 ± 270.00 ^a^	100.00 ± 0.00 ^ab^	2135.00 ± 2001.11 ^a^	3699 ± 2003.83 ^a^	−4.50 ± 1.06 ^b^
Diet 3	385.33 ± 214.50 ^cd^	96.67 ± 66.58 ^ab^	−330.00 ± 523.26 ^b^	1411 ± 4.44 ^c^	−3.53 ± 0.47 ^ab^
Diet 4	276.50 ± 2.12 ^d^	53.33 ± 20.82 ^b^	−250.00 ± 494.97 ^b^	683 ± 131.55 ^c^	−2.72 ± 0.41 ^a^
Megabee	475.67 ± 45.96 ^bc^	60.00 ± 34.64 ^b^	−160.00 ± 545.25 ^b^	1483 ± 637.01 ^c^	−3.07 ± 0.64 ^a^
Control	-	-	−375.00 ± 459.62 ^b^	1812 ± 339.15 ^bc^	−2.61 ± 0.27 ^a^

Different letters represent significant differences at *p* < 0.05 based on Duncan’s multiple range comparisons (DMRTs), and the results are presented as the means and standard deviations.

## Data Availability

The data that are presented in this study are available in the article and Supplemental information.

## Supplementary Materials

The following supporting information can be downloaded at: https://www.mdpi.com/article/10.3390/insects15020101/s1, Table S1: Primer information.
