# Supplementary material for: Comparative Study of the Effect of Pollen Substitute Diets on Honey Bees during Early Spring
Source: Insects. 2024 Feb 1;15(2):101. doi: 10.3390/insects15020101 (PMC10889207; doi:10.3390/insects15020101)
Supplement: Supplementary file 1 [file insects-15-00101-s001.zip › insects-2835349-supplementary.pdf]

Table S1. Primer information

Gene		Primer sequence (5’-3’)	Size (bp)
DWV	F	CGAAACCAACTTCTGAGGAA	174
	R	GTGTTGATCCCTGAGGCTTA	
Vg	F	GTTGGAGAGCAACATGCAGA	150
	R	TCGATCCATTCCTTGATGGT	
ß-actin	F	AGGAATGGAAGCTTGCGGTA	181
	R	AATTTTCATGGTGGATGGTGC	
